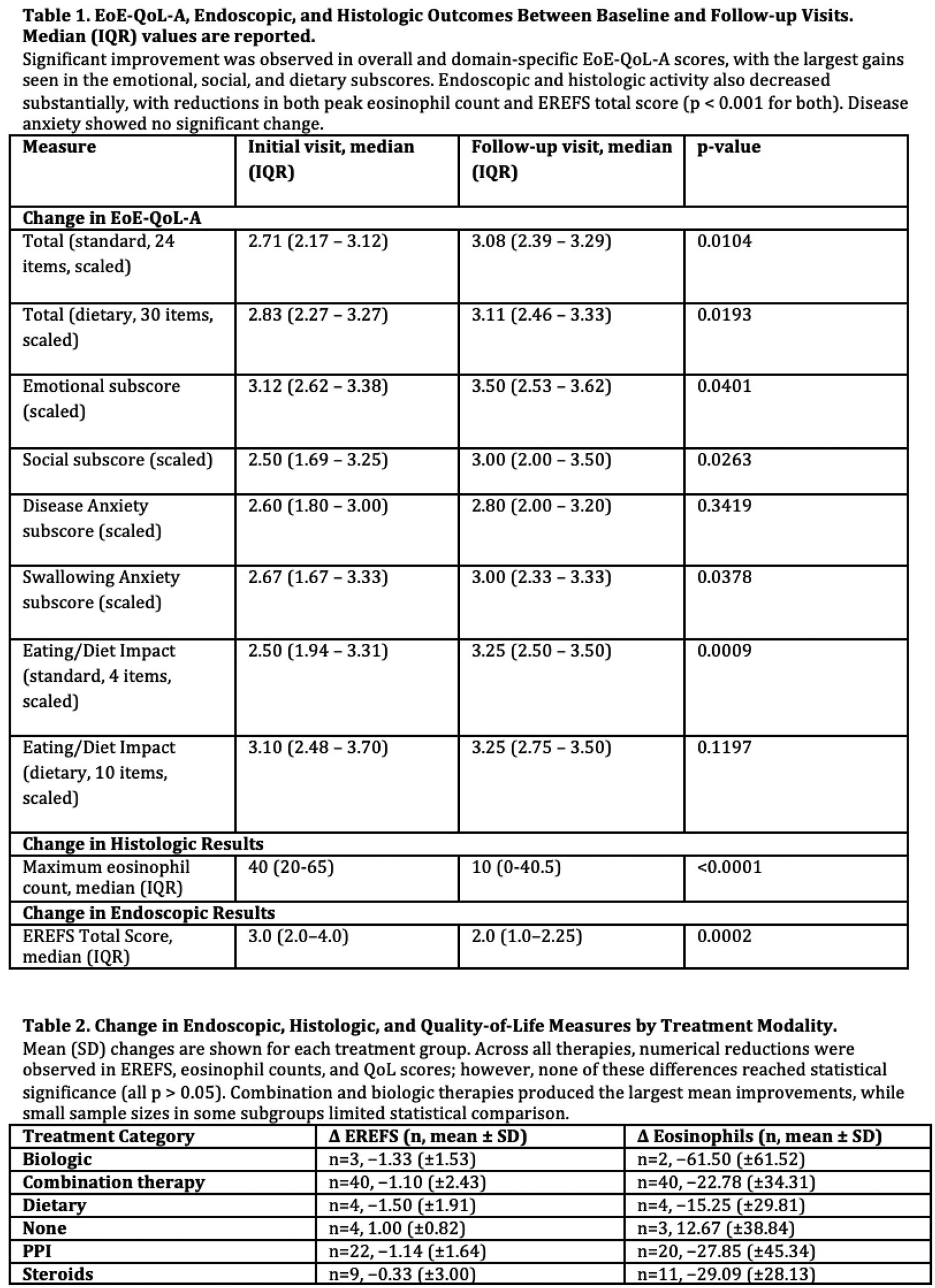# Poster Session II - A189 A PROSPECTIVE APPROACH TO TREATMENT RESPONSE IN EOSINOPHILIC ESOPHAGITIS IN ALBERTA

**DOI:** 10.1093/jcag/gwaf042.189

**Published:** 2026-02-13

**Authors:** P Dhingra, M Quon, G Tadros, A Herik, J Ha, D Kim, R Dhaliwal, I Stukalin, M Buresi, D Y Li, M Woo, E Mak, C Ma, M Gupta

**Affiliations:** University of Alberta, Edmonton, AB, Canada; University of Calgary Cumming School of Medicine, Calgary, AB, Canada; University of Calgary Cumming School of Medicine, Calgary, AB, Canada; University of Calgary Cumming School of Medicine, Calgary, AB, Canada; University of Calgary Cumming School of Medicine, Calgary, AB, Canada; University of Calgary, Calgary, AB, Canada; University of Calgary, Calgary, AB, Canada; Medicine, University of Calgary, Calgary, AB, Canada; University of Calgary, Calgary, AB, Canada; University of Calgary, Calgary, AB, Canada; University of Calgary, Calgary, AB, Canada; University of Calgary, Calgary, AB, Canada; University of Calgary, Calgary, AB, Canada; Medicine, University of Calgary, Calgary, AB, Canada

## Abstract

**Background:**

Eosinophilic esophagitis (EoE) is a chronic, immune-mediated inflammatory disorder of the esophagus characterized by dysphagia, food impaction, and progressive fibrostenotic remodeling if untreated. Diagnosis is based on symptoms of esophageal dysfunction, endoscopic findings, and histopathologic criteria of ≥ 15 eosinophils per high-power field. Despite advances with proton pump inhibitors (PPIs), topical steroids, elimination diets, biologics, and dilation, long-term outcomes and their impact on quality of life (QoL) remain unclear.

**Aims:**

To (1) evaluate real-world treatment utilization and longitudinal response in a Canadian EoE cohort, (2) assess changes in histologic, endoscopic, and QoL outcomes following therapy, and (3) examine relationships between objective disease activity and patient-reported outcomes.

**Methods:**

Adults (≥18 years) with confirmed EoE followed at a tertiary center in Alberta between 2011–2025 were prospectively included. Data on demographics, and clinical, endoscopic, histologic, and QoL data were collected. Endoscopic severity was measured using the EoE Endoscopic Reference Score (EREFS), histologic activity by peak eosinophil count (cells/HPF), and QoL using the Adult EoE Quality of Life Questionnaire (EoE-QOL-A). Paired analyses were conducted with the Wilcoxon signed-rank test, correlations with Spearman’s ρ, and between-group comparisons with Kruskal-Wallis test.

**Results:**

Seventy-seven patients were analyzed (median age 30.4 years with IQR 17.8-36.2 years; 76.7% male; median disease duration 6.2 years). Most received PPIs (90%), topical steroids (73%), dietary therapy (53%), or combination regimens (50%). Median eosinophil counts declined from 40 to 10 eos/HPF (*p* < 0.0001), and median EREFS improved from 3.0 to 2.0 (*p* = 0.0002), reflecting significant histologic and endoscopic improvement. Total EoE-QOL-A scores improved from 43 to 24 (*p* < 0.0001), with gains across emotional, social, dietary, and anxiety domains (Table 1). However, changes in eosinophil count and EREFS were not correlated with QoL improvement (ρ = 0.14 and –0.20 respectively, *p* > 0.1). Outcomes did not differ significantly by treatment type for changes in eosinophils (*p* = 0.37), EREFS (*p* = 0.33), or QoL (*p* = 0.59), though combination therapy demonstrated numerically greater overall improvement (Table 2).

**Conclusions:**

In this real-world prospective cohort, treatment for EoE resulted in substantial improvement in histologic, endoscopic, and QoL outcomes, though outcomes did not differ by treatment class. The lack of correlation between objective markers and patient-reported outcomes highlight the multidimensional nature of EoE and the need to integrate QoL assessments into clinical decision-making.

**Funding Agencies:**

None